# Common Errors in Diagnosis: The Pitfalls of Panel and Private Practice

**Published:** 1913-11-08

**Authors:** F. J. Smith

**Affiliations:** Physician to the London Hospital, etc.


### COMMON ERRORS IN DIAGNOSIS.

### The Pitfalls both of Panel and Private Practice.

By F. J. SMITH, M.D., F.R.C.P., Physician to the London Hospital, etc.

(A Lecture delivered at the Polyclinic.)

He who boasts that he has never made a mistake must be either very young—too young to have had much experience—or a liar, or a fool. I could justify these terms by my own experience, but my experience has made me too humble to wish to give similar pain to others.

The origin of mistakes may be gross ignorance, or the minor form of that malady known as inexperience. Or it may be over-confidence in one's infallibility. More frequently mistakes arise from carelessness and an incomplete survey of the situation; and they may really arise from over-pressure of work, a cause which is liable to be multiplied by recent developments in club practice in connection with the National Insurance Act. It is not fair to class as a mistake an error made after a full and careful examination of a case, as the most experienced and careful, even when assisted by the sciences which are ancillary to medicine, may make a sin of omission or one of commission. The effect on the practitioner of having made a mistake should be a determination not to be caught napping again.

### Managing Patients' Friends.

Mistakes may arise in diagnosis, in prognosis, or in treatment, the latter term being held to include the handling of the patient and his friends, for the management of the friends by the doctor may need to be more delicate and careful than that of the patient. A mistake may be made even before the patient is seen by the doctor, when he receives the summons to see the patient. The way to avoid a mistake here is to attend to all urgent summonses or have a quite legitimate professional reason for not doing so. In connection with urgent summonses the two things which occur to one are poison and hæmorrhage.

The next opportunity for mistake arises when the patient is present. It may be a tactical mistake; an attitude may be adopted, or words said, which may be resented at the very outset. In cases of suspected malingering the doctor may too early allow his suspicion to be evident, and so defeat any object sought by the interview. The liability to this is especially great in panel practice and among the very poor, who usually have a very keen sense of sympathy or antipathy.

With regard to mistakes in diagnosis, the first mistake may arise from impatience in listening to the patient's account of symptoms and history of the illness. Tact and experience alone can differentiate between garrulous imbecility and valuable information. Don't shut up the source of information too soon, or you may lose the very point which would put you right. A man fell downstairs while carrying something, and complained of physical and nervous disability in the hand and arm, which were also definitely wasted. He was demonstrated as a case of injury, but later it transpired that he had had infantile paralysis. Diagnosis should be considered under two categories—the preliminary, and the later one, which is made more at leisure, with the patient in bed in his own home, and when there has been time for watching and considering the case. The mistakes which matter are more frequent in the first of these categories.

### "Mala Praxis."

On an ordinary night at one's surgery 95 per cent. of the cases—excluding cases of accident—complain of one or more of the following: Pain, cough (including shortness of breath), upset of alimentary functions, headache, or a vague feeling of seediness. In these carelessness will be the main reason for failure. Answers to questions as to the seat, character, and duration of pain, for instance, will throw much light on the case. If the pain be in a limb, never allow the word "rheumatism" to be used by the patient without a close cross-examination of what he means by it, and without insisting on seeing the place to which it is referred. On various occasions I have been asked to see cases stated to be rheumatism—and in some the doctor has acquiesced in the patient's diagnosis—and have found pus round the ankle joint, acute epiphysitis, sarcoma, osteo-myelitis—all simply because a pain in the limb was complained of. In one case which was said to be rheumatism I even found an aneurysm of the popliteal artery. If the seat of the pain be the abdomen, it must be carefully examined, and an attempt made to estimate its cause and origin. To allow a patient to come and say he has got a belly-ache, to give him a bottle of medicine and say he will be better in the morning, without examining further, is sheer *mala praxis*; it is fraud without any excuse. Similarly, if the pain complained of is in the chest, the exact diagnosis is matter for subsequent thought and examination. There should be at least a cursory stethoscopic examination to determine the question not of pleura *versus* diaphragm, but bedroom *versus* business; of a boil or carbuncle *versus* perforating empyœma; and all these *versus* some acute internal chest trouble.

Headaches and general seediness, constipation and diarrhœa, these alimentary upsets at least demand one precautionary measure—namely, observation of the temperature, and this even at the busiest time. A rise or a fall of temperature imperatively demands that the case shall be treated seriously, and sent home to bed. The preliminary diagnosis goes no further.

### Incautious Admissions.

More mistakes are made in professional matters in connection with what the doctor says, and his demeanour in saying it, than in any other respect. "Familiarity breeds contempt," and the doctor is apt lightly to make admissions and statements which the patient and his friends hang upon and treasure up, and which the unfortunate practitioner may subsequently find have been perverted and misused to his detriment. In the case of tubercular meningitis, for instance, how often is the preliminary vomiting ascribed to the eating of something unsuitable? Then the casual remark is made, "Give the child a dose of this, and it will be all right in the morning." The doctor may hear of the remark later, "Doctor So-and-So said my child would be all right next day; I buried him a week after."

An abdominal pain with diarrhœa may mean typhoid, or some gross disease of the alimentary canal; so you should say: "This requires more attention than I can give it now; take this medicine and stay at home, and I will come and have a look at you to-morrow." The great mistake is to say too much too soon. It is wiser to say nothing voluntarily after examining the patient, especially if you suspect that serious trouble may be lurking behind an innocent superficial appearance.

### Mistakes in Prognosis.

There comes the inevitable conclusion of the interview, when it is certain you will be asked about the general lines on which the patient must be handled. Must he go to bed? Must he stay indoors, or may he go to work? And questions as to food and drink are fairly sure to be asked, and there are many ways and tones of voice in which these can be answered without giving offence. These matters make a great deal of difference as to the practitioner's reputation and the degree of confidence he inspires. When the patient's illness takes on the more serious turn, let him remember that your demeanour and words prepared him for it.

Mistakes in prognosis are constantly being made —at any rate in the practitioner's own mind—and the only way to avoid unpleasant results is to avoid giving a forecast on limits which are too narrow; don't be too rigid and too absolute. None of us have a sufficient knowledge to enable us to assess accurately the endurance of life in illness, and persons differ very widely. We do not know enough about the dynamics of the functions of the body. In our own minds we base our prognosis on the law of averages, which is gained by reading and experience, and tempered by the peculiar features of our case. The only way to avoid unpleasant results of mistakes is to be nearly as frank with the friends of the patient as we are with ourselves; but here again don't say too much too soon. When you have made a careful diagnosis, do not give a prognosis if you can avoid it; keep the star of hope shining as long as possible.

### Digitalis Poisoning.

Mistakes in treatment are of two or three varieties. The first variety is that of giving a gross overdose; it may be an accident, or there may be wrong labels, and these will always occur to some extent. Ordinary mistakes in treatment arise directly out of mistakes in diagnosis. One variety of mistake arises out of an imperfect acquaintance with the power of drugs in varying dosage. For instance, quinine is said to have a tonic effect, and possibly it has when given in doses of one or two grains. But it is chiefly used for its specific effects in malaria, and then it is a mistake to give it in doses of less than ten grains. With regard to bismuth, I would say it is a mistake to give bismuth to beefsteaks—*i.e.*, to administer it while there is food in the stomach. It will get on to the food instead of on to the stomach. With regard to the giving of digitalis, please remember it is a mistake to give digitalis without watching the secretion of urine, and, if possible, measuring the amount passed.

### Opium as a Tonic.

Doubtless the grave hides many such mistakes, the death being certified as due to morbus cordis, not digitalis poisoning. And a last word as to opium and morphia. It is a mistake to think that in small medicinal doses these are cardiac depressants. I cannot think why that mistake has arisen. In ordinary doses morphia and opium are not only not depressants—they are deliberate cardiac tonics. And if they will, without much alimentary upset, give the patient a good night's rest, there is not only the tonic effect of the drug, but that of a good night's rest also. If you are given the choice of one drug for a case of bad heart choose first magnesium sulphate; and secondly choose opium; and thirdly digitalis, if the patient can rest in bed.

